# GDF15 Improves Renal Injury Induced by Ectopic Lipid Deposition via AMPK/SIRT1 Pathway-Mediated Autophagy

**DOI:** 10.3390/metabo16050336

**Published:** 2026-05-18

**Authors:** Qiang Zhang, Xidong Yang, Yuxuan Yang, Min Wang, Yulin Wu, Xin Xie, Yongjun Jin, Ming Yang, Meizi Yang

**Affiliations:** 1 Department of Pharmacology, Binzhou Medical University, Yantai 264003, China; zhangziqiang0304@163.com (Q.Z.); 17769237673@163.com (Y.W.); xiexin_phd@163.com (X.X.); 2 Department of Pharmacy, Yanbian University, Yanji 133000, China; pharmdong@126.com; 3 Department of Endocrinology, The Second Clinical Medical College, Binzhou Medical University, Yantai 264003, China; yuxuanyang0112@163.com (Y.Y.); wangmin_endocrine@163.com (M.W.)

**Keywords:** GDF15, obesity, autophagy, AMPK/SIRT1, kidney, Ectopic lipid deposition

## Abstract

**Highlights:**

**What are the main findings?**

• High expression of GDF15 is closely associated with an obesity-resistant phenotype and contributes to alleviating obesity-induced renal ectopic lipid deposition and kidney injury.
• Knockdown of GDF15 receptor GFRAL significantly exacerbated the obese phenotype in mice, accompanied by a reduction in autophagy levels, thereby leading to increased renal lipid deposition and aggravated kidney injury.

**What are the implications of the main findings?**

• GDF15 possesses the dual effects of reducing body weight and protecting the kidneys, holding promise as a potential therapeutic target for obesity and its associated nephropathy and providing a new strategy for its clinical prevention and treatment.

**Abstract:**

Objectives: Obesity precipitates excessive lipid accumulation within the kidney, culminating in ectopic lipid deposition that compromises target organ function through lipotoxicity. Given the pivotal role of GDF15 in lipid metabolism, this study aims to determine whether GDF15 can ameliorate ectopic lipid deposition and mitigate the resulting renal injury. Methods: C57BL/6J mice were used to establish a high-fat diet-induced obesity model. Based on Lee’s index, the mice were categorized into a diet-induced obesity group and an obesity-resistant group. Subsequently, the diet-induced obesity group received an injection of AAV-shGFRAL to knock down the GFRAL receptor. Results: In obesity resistant mice, ectopic lipid deposition in the kidneys was markedly reduced, accompanied by decreased expression of the renal injury marker KIM-1 and significantly elevated levels of GDF15. Modulation of the GDF15-GFRAL axis demonstrated that reduced autophagy levels led to increased lipid accumulation and exacerbated renal injury. Conversely, GDF15 activates the AMPK/SIRT1 signaling pathway to promote cellular autophagy, thereby mitigating renal damage induced by ectopic lipid deposition. Consistent with this mechanism, the suppression of autophagy results in the aggravation of renal injury caused by ectopic lipid accumulation. Conclusions: GDF15 ameliorates renal injury induced by ectopic lipid deposition in the kidney primarily through activation of autophagy via the AMPK/SIRT1 signaling pathway.

## 1. Introduction

The primary cause of obesity-related glomerulopathy (ORG) is adiposity. Ectopic lipid deposition caused by obesity serves as a key trigger that induces kidney damage and drives the deterioration of renal function through lipotoxic effects [1]. When energy intake exceeds energy expenditure, most of the ingested energy is stored in adipose tissue in the form of triglycerides (TGs). Excess lipids may enter non-fat tissues such as the kidneys and liver, forming ectopic lipid deposition (ELD) [2,3,4,5,6]. When lipid deposition occurs in the kidneys, lipid breakdown in renal endothelial cells, podocytes, and proximal tubular epithelial cells decreases, leading to lipotoxic effects and causing kidney damage. This leads to pathological characteristics such as glomerular hypertrophy, renal interstitial fibrosis, and inflammation, exacerbating renal dysfunction [7,8]. ELD has gradually become an important and independent risk factor for obesity-related glomerulopathy. Therefore, improving ectopic lipid deposition in the kidneys will reduce the lipotoxicity caused by lipid accumulation, thereby delaying the onset and progression of ORG. Studies have shown that in the early stage of ELD, the lipolytic products or metabolic derivatives of free fatty acids (FFA) transfer fatty acids from lipid droplets to mitochondria by activating autophagy, promoting ATP production, and thereby regulating lipid metabolism and energy homeostasis. This improves ectopic lipid deposition and reduces lipotoxicity [9,10]. Targeting autophagy to regulate the process of ELD may be an effective strategy for treating obesity and obesity-related metabolic diseases.

Growth differentiation factor 15 (GDF15), as a stress responsive cytokine, is involved in the occurrence and development of diseases such as obesity, tumors, and non-alcoholic fatty liver disease. Studies have shown that GDF15 is associated with anorexia in patients with advanced cancer [11], and GDF15 may be related to inhibiting food intake and reducing body weight. The identification of the GDF15-GFRAL axis provides a new interpretation of GDF15’s role in reducing body weight, which is mediated by the inhibition of the feeding center [12,13,14].

Previous studies have found that knocking down the GDF15 gene accelerates the progression of diabetic nephropathy and other complications in diabetic rats [15]. In summary, GDF15 may have a potential protective effect on renal damage caused by ectopic lipid deposition and is expected to become a new target for the treatment of obesity-related nephropathy. AMP-activated protein kinase (AMPK), as a key regulatory factor that initiates autophagy, is an important kinase involved in cellular energy sensing and cell signaling regulation during autophagy. Its activity can activate the transcription of autophagy-related genes through multiple pathways, promoting the onset and progression of autophagy [16]. GDF15 can induce a series of biological effects by regulating AMPK phosphorylation, including promoting fatty acid oxidation, improving insulin resistance, and inhibiting inflammatory responses [17]. The regulation of renal autophagy by GDF15 is likely mediated by the AMPK pathway. Studies have shown that AMPK phosphorylation activates SIRT1, initiates autophagy responses [18], and regulates energy homeostasis and metabolic stress [19]. The above research suggests that GDF15 may act on the AMPK/SIRT1 pathway to increase autophagy levels and improve renal ELD.

This study aims to reveal the impact of GDF15 on renal ELD and autophagy levels, and to elucidate the mechanism by which GDF15 regulates ELD to improve obesity-related glomerulopathy based on autophagy, providing intervention targets for alleviating ectopic lipid deposition and preventing ORG.

## 2. Materials and Methods

### 2.1. Animals

Male C57BL/6J mice (Jinan Peng Yue Experimental Animal Breeding Co., Ltd., Jinan, China) were used to establish the obesity model. All animal experiments were approved by the Animal Ethics Committee of Binzhou Medical University and strictly performed in accordance with guidelines for biomedical research ethics. Four-week-old mice (14–16 g) were randomly divided into a normal chow control group (NC) and a high-fat diet-induced obesity model group (HFD) using a random number table, with no significant difference in initial body weight between groups. The NC group was fed a standard chow diet, while the HFD group received a high-fat diet. After 8 weeks of high-fat feeding, mice that failed to develop obesity based on Lee’s index were selected from the HFD group and designated as diet-induced obesity-resistant (DIO-R) mice. Mice that met the criteria for obesity were then randomly allocated into DIO, shControl, and shGFRAL groups, with no significant differences in body weight among these groups at baseline. The shGFRAL group received a stereotactic injection of adeno-associated virus (AAV), whereas the shControl group was injected with an empty vector. All mice were housed under controlled temperature and humidity conditions (22 ± 2 °C and 55 ± 5%, respectively) on a 12/12 h light/dark cycle, with free access to food and water.

Adeno-associated virus carrying the expression sequence CCGGGTGACTCCTGCAAGATAAATCTCGAGATTTATCTTGCAGGAGTCACCTTTTTTG was injected to inhibit the expression of the GDF15 receptor GFRAL (shGFRAL group). The empty vector virus was used as a control (shControl group) with the expression sequence ACCGCCTAAGGTTAAGTCGCCCTCGCTCGAGCGAGGGCGACTTAACCTTAGGTTTTTTG (OBiO Technology Corp., Ltd., Shanghai, China). Mice received bilateral injections of 0.8 μL of AAV-shGFRAL or AAV-shControl over a period of 3–10 min. The stereotactic coordinates were 7.32 mm posterior to Bregma, 0.025 mm lateral to the midline, and 4.5 mm ventral to the skull surface.

From the 5th week, the body weight, body length, and anal temperature of the mice were measured until the 17th week. Body length was defined as the distance from the tip of the nose to the anus. The Lee index was calculated using the following formula: body weight (g) ^1/3^ × 10^3^/length (cm).

### 2.2. Oil Red O Staining

Tissues were embedded and sectioned at a thickness of 10 μm using a cryostat. The frozen sections were stained using a Modified Oil Red O Staining Kit (Beyotime, Shanghai, China) for 10–20 min. For cells, samples were fixed with 4% paraformaldehyde for 10 min and stained with Oil Red O solution for 10–20 min, followed by washing with PBS. All samples were subsequently observed under a microscope (Olympus, Tokyo, Japan).

### 2.3. Hematoxylin and Eosin (H&E) Staining

Tissues were fixed with 4% PFA, embedded in paraffin, and sectioned into 4-μm-thick slices using a microtome. The sections were then stained with H&E and observed under a microscope.

### 2.4. PAS Staining

Following paraffin sectioning, tissues were rehydrated, washed in distilled water, and then rinsed with tap water for 2–3 min. PAS staining was performed using a kit (Solarbio, Beijing, China), and the sections were examined under a microscope.

### 2.5. Western Blotting

Renal tissue homogenates and HK-2 cell suspensions were prepared, and protein concentrations were quantified using the bicinchoninic acid (BCA) assay (Beyotime Biotechnology, Shanghai, China). The samples were separated by SDS-PAGE and subsequently transferred onto PVDF membranes. After blocking, the membranes were incubated overnight with the following primary antibodies: anti-GDF15 (Proteintech, Wuhan, China), anti-KIM-1 (Proteintech, Wuhan, China), anti-PLIN2 (Proteintech, Wuhan, China), anti-FAT/CD36 (Affinity Biosciences, San Francisco, CA, USA), anti-LC3 (Proteintech, Wuhan, China), anti-p62 (Proteintech, Wuhan, China), anti-GAPDH (Proteintech, Wuhan, China), anti-AMPK (ELK Biotechnology, Wuhan, China), anti-phospho-AMPK (Immunoway, Houston, TX, USA), and anti-SIRT1 (Immunoway, Houston, TX, USA). Then the membranes were incubated with appropriate secondary antibodies, and band intensities were analyzed using ImageJ software 1.54.

### 2.6. Enzyme-Linked Immunosorbent Assay (Elisa)

ELISA kits (Bioswamp Life Science Lab., Wuhan, China) were used to measure the concentrations of GDF15, KIM-1, CPT-1, and insulin in serum, as well as CPT-1 levels in HK-2 cells, in accordance with the manufacturer’s protocols.

### 2.7. Biochemical Assay

The levels of free fatty acids (Abbkine, Wuhan, China), creatinine (Cre) (Nanjing Jiancheng, Nanjing, China), blood urea nitrogen (BUN) (Nanjing Jiancheng, Nanjing, China), and glucose (Abbkine, Wuhan, China) in serum were measured using assay kits.

### 2.8. Quantitative Real-Time PCR (q-PCR)

Total RNA was extracted from brain tissues by precisely dissecting the AP and NTS regions. Approximately 60–120 mg of tissue was homogenized using TRIzol reagent. After centrifugation, the supernatant was collected and mixed with one-fifth volume of chloroform for phase separation. The aqueous phase containing RNA was precipitated with an equal volume of isopropanol. The RNA pellet was washed with 75% ethanol, air-dried, and dissolved in nuclease-free water. Quantitative reverse transcription was performed, followed by q-PCR analysis using a commercial kit (Lablead, Beijing, China).

### 2.9. Cell Culture

HK-2 cells (Procell, Wuhan, China) were cultured in complete medium containing 10% fetal bovine serum and 1% penicillin/streptomycin at 37 °C with 5% CO_2_. When the cells reached 60–70% confluence, the following treatment groups were established: HL group (induced with 250 μmol/L sodium oleate and 125 μmol/L sodium palmitate for 48 h); GDF15 group (treated with 100 ng/mL GDF15 for 48 h, following HL induction); and GDF15 + 3-MA group (treated with 5 mM 3-MA for 18 h, subsequent to GDF15 treatment). In the inhibition experiment, cells were treated with either 10 μM Compound C or 1 μM EX527 for 24 h. Cells were subsequently observed or harvested for further analysis.

### 2.10. Immunofluorescence (IF)

Cells grown on culture slides in 24-well plates were fixed with 4% paraformaldehyde for 15 min upon reaching the target density. Subsequently, they were permeabilized with 0.5% Triton X-100 (Sigma-Aldrich, St. Louis, MO, USA) and blocked with 10% goat serum. The slides were then incubated overnight at 4 °C with primary antibodies against LC3 (Proteintech; 1:250) and SIRT1 (Immunoway; 1:200). After washing, the slides were incubated with appropriate secondary antibodies (protected from light) and the nuclei were counterstained with DAPI. Finally, the slides were mounted and visualized using an inverted fluorescence microscope.

### 2.11. Cell Viability Assay

Cell viability was assessed using a CCK-8 kit (Meilunbio, Dalian, China) following induction with oleic acid/sodium palmitate.

### 2.12. Statistical Analysis

All experimental data were subjected to statistical analysis and graphed using GraphPad Prism software (version 10.2.3, GraphPad Software, San Diego, CA, USA). Comparisons between groups were conducted using an unpaired *t*-test and one-way ANOVA. *p*-value < 0.05 was considered statistically significant.

## 3. Results

### 3.1. Elevated GDF15 Levels Are Associated with Reduced Renal Lipid Deposition and Ameliorated Renal Injury in Diet-Induced Obesity-Resistant Mice

**Figure 1:**
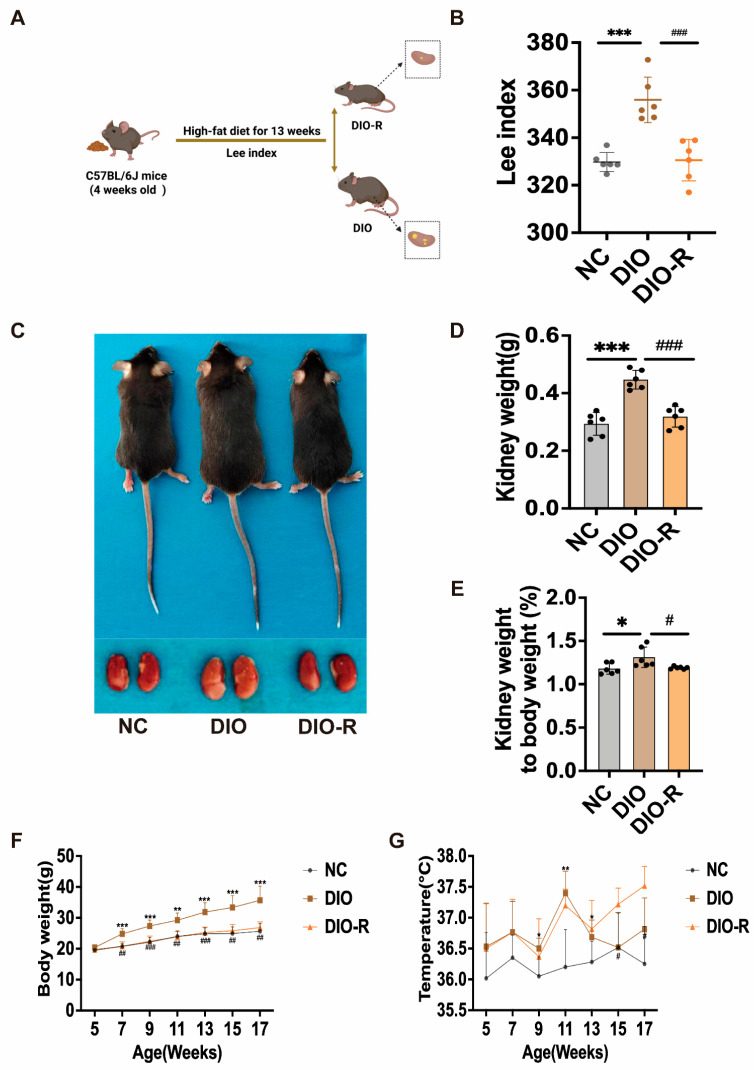
Analysis of phenotypic characteristics in diet-induced obese and obesity-resistant mice. (**A**) Establishment of the animal model. (**B**) Lee index (*n* = 6). (**C**) Mouse body size and kidney appearance. (**D**) Kidney weight (n = 6). (E) Ratio of kidney weight to body weight (*n* = 6). (**F**) Body weight change curve (*n* = 6). (**G**) Temperature change curve (*n* = 6). Mean ± SD. * *p* < 0.05, ** *p* < 0.01, and *** *p* < 0.001 vs. the NC group; ^#^ *p* < 0.05, ^##^ *p* < 0.01, and ^###^ *p* < 0.001 vs. the DIO group.

**Figure 2:**
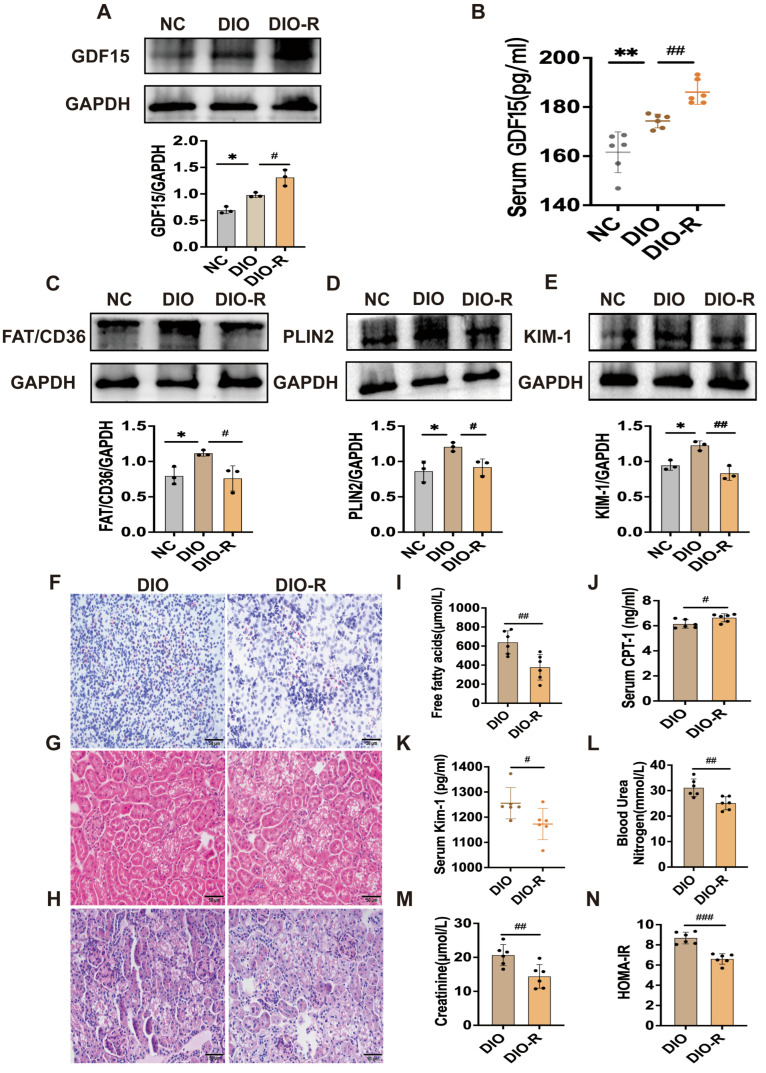
GDF15 ameliorates renal ectopic lipid deposition and injury in obesity-resistant mice. (**A**) GDF15 protein expression in kidneys (*n* = 3). (**B**) Serum GDF15 levels (n = 6). (**C**–**E**) Quantification of FAT/CD36, PLIN2, and KIM-1 protein expression in kidneys (*n* = 3). (**F**–**H**) Oil Red O, H&E, and PAS staining of renal tissues (Scale bar = 50 μm). (**I**) Serum free fatty acid (FFA) (*n* = 6). (**J**) Serum CPT-1 (*n* = 6). (**K**–**M**) Serum levels of KIM-1, blood urea nitrogen (BUN), and creatinine (Cre) (*n* = 6). (**N**) Homeostatic model assessment of insulin resistance (HOMA-IR) (*n* = 6). Mean ± SD. * *p* < 0.05 and ** *p* < 0.01 vs. the NC group; ^#^ *p* < 0.05, ^##^ *p* < 0.01, and ^###^ *p* < 0.001 vs. the DIO group.

Compared to the DIO group, the DIO-R group showed significantly lower body weight, kidney weight, and kidney index, with increased body temperature (Figure 1). Interestingly, our investigation revealed a statistically significant elevation in GDF15 concentration in both renal tissue and serum of DIO-R mice, accompanied by a significant reduction in HOMA-IR (Figure 2A,B,N). In DIO-R mice, the overexpression of CPT-1 was accompanied by significant decreases in the expression levels of FAT/CD36 and PLIN2, as well as reduced FFA concentrations, indicative of attenuated renal ectopic lipid deposition (Figure 2C,D,I,J). Oil Red O staining further corroborated a significant reduction in lipid droplet accumulation in the renal tissue of the DIO-R group (Figure 2F). These findings demonstrate that the elevated GDF15 levels in DIO-R mice are closely associated with alleviated renal ectopic lipid deposition. Meanwhile, the expression of KIM-1 in renal tissue was significantly reduced (Figure 2E), and the serum concentrations of KIM-1, BUN, and Cre were also significantly lower (Figure 2K,L,M). Histological evaluation using HE and PAS staining demonstrated that the renal morphology and structure of DIO-R mice tended to normalize, with no evident glomerular hypertrophy or mesangial matrix expansion observed (Figure 2G,H). These findings suggest that elevated GDF15 levels in the DIO-R group are associated with a mitigation of renal injury.

### 3.2. The Impact of Blocking the GDF15-GFRAL Axis on Glycolipid Metabolism and Renal Function in Obese Mice

**Figure 3:**
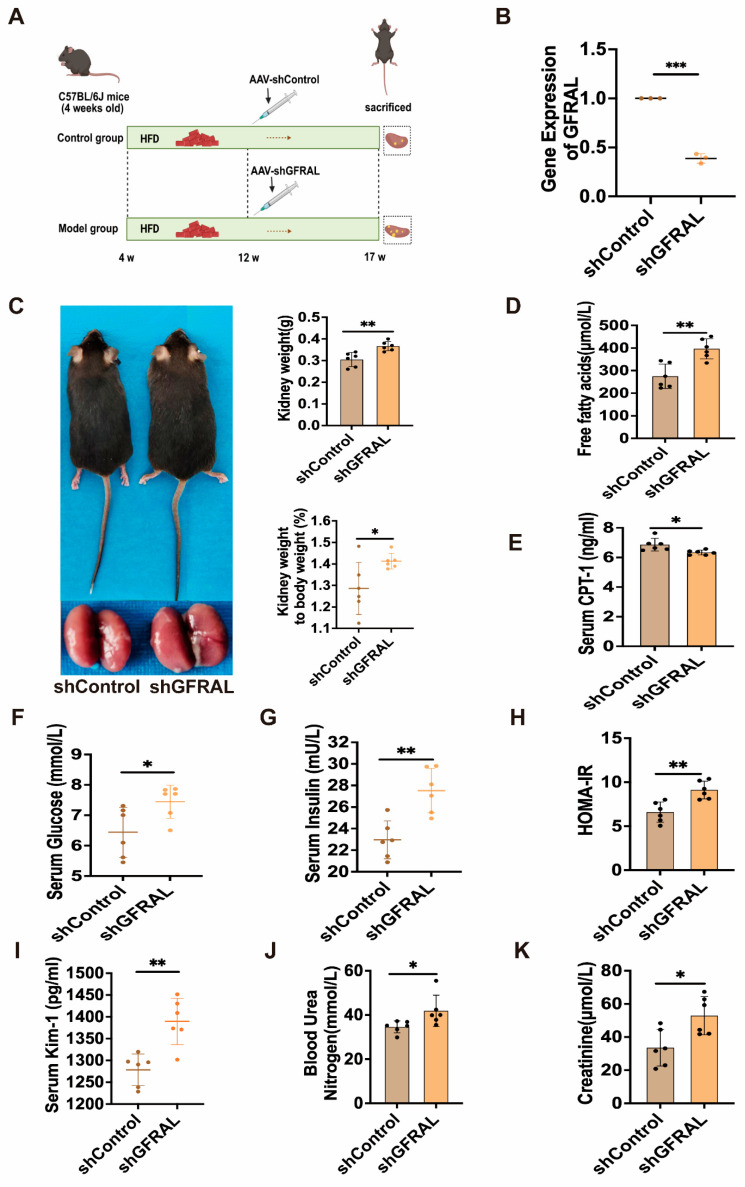
Blockade of the GDF15-GFRAL pathway induces glucose and lipid metabolic disorders and renal dysfunction in mice. (**A**) Schematic diagram of the animal grouping and treatment protocol. (**B**) GFRAL mRNA expression levels (*n* = 3). (**C**) Mouse body size, kidney appearance, kidney weight (*n* = 6), and percentage of kidney weight to body weight (*n* = 6). (**D**,**E**) Serum free fatty acids (FFA) and CPT-1 levels (*n* = 6). (**F**,**G**) Serum glucose and insulin levels (*n* = 6). (**H**) HOMA-IR (*n* = 6). (**I**–**K**) Serum levels of KIM-1, blood urea nitrogen (BUN), and creatinine (Cre) (*n* = 6). Mean ± SD. * *p* < 0.05, ** *p* < 0.01, and *** *p* < 0.001 vs. the shControl group.

To further elucidate the link between GDF15 and obesity-induced renal injury, we knocked down GFRAL expression in the AP and NTS of DIO mice via stereotaxic injection of AAV-shGFRAL. Knockdown efficiency was confirmed by qRT-PCR (Figure 3B). Inhibition of the GDF15-GFRAL axis resulted in a significant increase in body weight, kidney weight, and ratio of kidney to body weight in the shGFRAL group (Figure 3C). Concurrently, elevated serum FFA levels and decreased CPT-1 levels suggested abnormalities in lipid metabolism (Figure 3D,E). Moreover, significant increases in blood glucose, insulin levels, and the HOMA-IR indicated disordered glucose metabolism (Figure 3F–H). Additionally, KIM-1, BUN, and Cre levels were significantly elevated in the serum (Figure 3I–K). Results demonstrated that knockdown of the GDF15-GFRAL axis exacerbates glucose and lipid metabolism disorders and induces renal injury in mice.

### 3.3. GFRAL Knockdown Promotes Renal Lipid Accumulation and Inhibits Autophagy to Induce Kidney Injury

**Figure 4:**
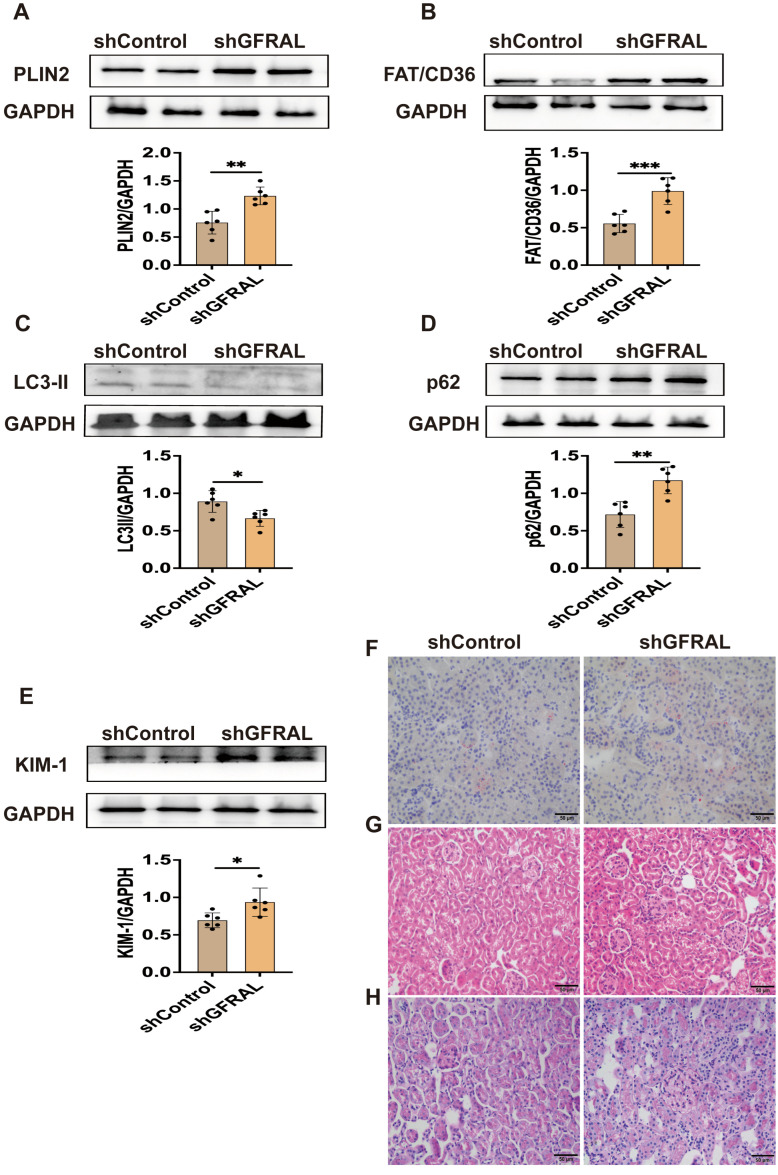
Effects of GDF15-GFRAL intervention on ectopic lipid deposition, autophagy, and kidney injury in mice. (**A**–**E**) PLIN2, FAT/CD36, LC3-II, p62, and KIM-1 protein levels in kidney tissue (*n* = 6). (**F**–**H**) Oil Red O, H&E, and PAS staining of renal tissues (Scale bar = 50 μm). Mean ± SD. * *p* < 0.05, ** *p* < 0.01, and *** *p* < 0.001 vs. the shControl group.

The expression of PLIN2 and FAT/CD36 in the kidneys was significantly elevated in the shGFRAL group, while LC3-II was reduced and p62 was increased, indicating the accumulation of lipids accompanied by a decrease in autophagic activity (Figure 4A–D). Concurrently, Kim-1 levels in renal tissues were significantly elevated (Figure 4E). Oil Red O, HE, and PAS staining further demonstrated substantial renal lipid deposition, glomerular hypertrophy, and mesangial matrix expansion in the shGFRAL group (Figure 4F–H). These findings indicate that renal injury induced by ectopic lipid accumulation may be associated with autophagy dysregulation.

### 3.4. GDF15 Promotes Autophagy to Ameliorate Lipid Deposition-Induced Injury in HK-2 Cells

**Figure 5:**
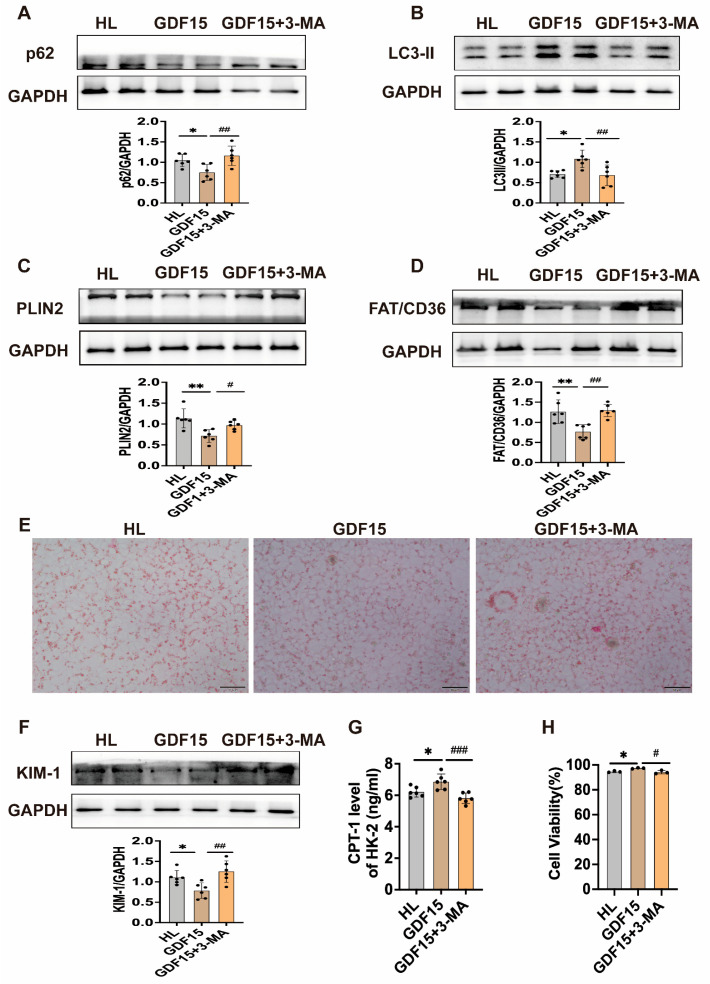
GDF15 attenuates ectopic lipid deposition-induced renal injury by promoting autophagy in HK-2 cells. (**A**–**D**) p62, LC3-II, PLIN2, and FAT/CD36 protein levels in HK-2 cells (*n* = 6). (**E**) Oil Red O staining (Scale bar = 50 μm). (**F**) KIM-1 protein levels in HK-2 cells (*n* = 6). (**G**) CPT-1 levels in HK-2 cells (*n* = 6). (**H**) Cell viability of HK-2 cells (*n* = 3). Mean ± SD. * *p* < 0.05 and ** *p* < 0.01 vs. the HL group; ^#^ *p* < 0.05, ^##^ *p* < 0.01, and ^###^ *p* < 0.001 vs. the GDF15 group.

GDF15 significantly enhances cellular autophagy levels (Figure 5A,B). Following the inhibition of autophagy, the CPT-1 level decreased and expression of PLIN2 and FAT/CD36 were significantly increased (Figure 5C,D,G). Oil Red O staining confirmed that GDF15 reduced intracellular lipid droplet accumulation (Figure 5E); however, autophagy inhibition attenuated the effect and exacerbated cell injury (Figure 5F,H). These findings indicate that GDF15 mitigates HL induced damage in HK-2 cells by activating autophagy and reducing lipid deposition.

### 3.5. GDF15 Regulates Autophagy Level via AMPK/SIRT1 Pathway

**Figure 6:**
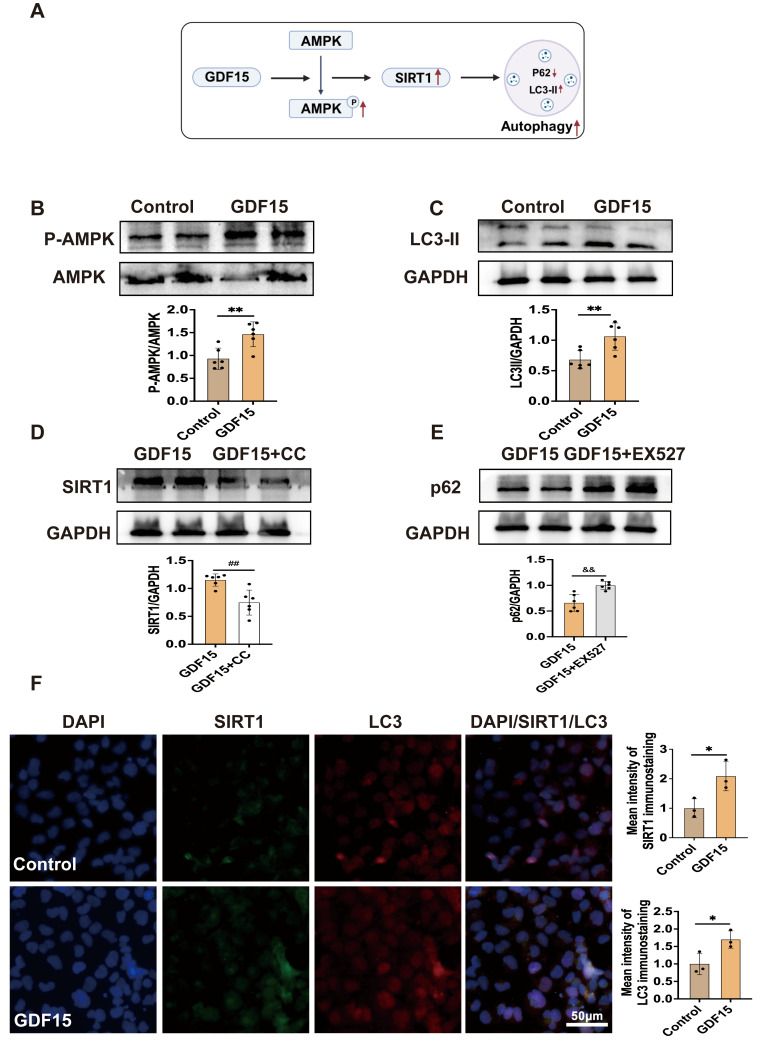
GDF15 regulates autophagy levels through the AMPK/SIRT1 signaling pathway in HK-2 cells. (**A**) Study design. (**B**,**C**) p-AMPK/AMPK and LC3-II levels in HK-2 cells (*n* = 6). (**D**) SIRT1 levels in HK-2 cells (*n* = 6). (**E**) p62 levels in HK-2 cells (*n* = 6). (**F**) Immunofluorescence staining of SIRT1 and LC3 (*n* = 3). Mean ± SD. * *p* < 0.05 and ** *p* < 0.01 vs. the Control group; ^##^ *p* < 0.01 vs. the GDF15 + CC group; ^&&^ *p* < 0.01 vs. the GDF15 + EX527 group.

To investigate the regulatory role of GDF15 in cellular autophagy, recombinant GDF15 protein was administered in cell culture experiments. The results demonstrated that GDF15 significantly promoted AMPK phosphorylation and induced SIRT1 expression, accompanied by an increase in autophagy levels (Figure 6B,C,F). Inhibition of AMPK phosphorylation led to a significant reduction in SIRT1 expression (Figure 6D). Furthermore, inhibition of SIRT1 resulted in a significant reduction in autophagy levels (Figure 6E). These findings demonstrate that GDF15 enhances cellular autophagy by activating the AMPK/SIRT1 signaling pathway.

## 4. Discussion

Obesity frequently presents with various metabolic syndromes, such as diabetes, non-alcoholic fatty liver disease, and obesity-related glomerulopathy (ORG). One of the core pathological features of ORG is lipotoxicity, which arises from ELD in the kidneys [20]. Among mice fed a high-fat diet, not all individuals develop diet-induced obesity (DIO); some mice exhibit obesity resistance (DIO-R) [21]. Moreover, DIO-R mice display significant differences from DIO mice in growth and biochemical parameters, as well as in the characteristics of ectopic lipid deposition. DIO-R mice exhibit lower Lee’s index, body weight, and relative kidney weight, along with an elevated body temperature, indicating that they may have a more active metabolic state. Notably, both renal and serum levels of GDF15, a key cytokine involved in metabolic homeostasis [22], were significantly higher in DIO-R mice than in DIO mice. The elevation of GDF15 is both a compensatory response to metabolic changes and the primary cause of obesity resistance. GDF15 mediates anorexia-like responses and ameliorates metabolic dysregulation by binding specifically to the GFRAL receptor localized in the nucleus tractus solitarius (NTS) and area postrema (AP) of the brain [23,24,25]. Accordingly, the high expression of GDF15 in DIO-R mice may be closely related to their metabolic phenotype and the mechanism underlying obesity resistance. This study further revealed that DIO-R mice exhibited high renal expression of GDF15, along with significantly reduced levels of ELD-related proteins FAT/CD36 and PLIN2, as well as the renal injury factor KIM-1. Given the inherent metabolic characteristics of obesity-resistant mice, multiple endogenous factors may collectively downregulate the expression of proteins related to lipid deposition. Our in vitro results indicate that GDF15 directly reduces the expression of FAT/CD36 and PLIN2 by activating autophagy. In vivo, inhibiting the central effects of GDF15 exacerbates the obese phenotype in mice and increases the renal expression of FAT/CD36 and PLIN2. This is consistent with the protective effect of GDF15 on the kidneys [26]. KIM-1 is a highly sensitive and specific biomarker for renal tubular injury, and its downregulation is widely recognized as a reliable indicator of alleviated tubular damage and improved renal function [27]. In the present study, reduced KIM-1 levels were accompanied by improved renal function, as reflected by decreased serum BUN, Cre and FFA levels. These observations collectively support the functional renoprotective effect of GDF15. Therefore, the endogenous upregulation of GDF15 may be a key mechanism by which DIO-R mice resist renal lipotoxicity and associated kidney injury induced by obesity.

Knockdown of GFRAL in the AP and NTS regions confirmed the critical regulatory role of the GDF15-GFRAL axis in murine metabolism and renal phenotypes. Following GFRAL knockdown, mice exhibited significant increases in body weight, kidney wet weight, and insulin resistance index, accompanied by elevated serum levels of FFA, BUN, and Cre. These findings indicate exacerbated metabolic dysregulation and renal injury, highlighting the contribution of the GDF15-GFRAL axis to central nervous system-mediated systemic energy homeostasis and peripheral organ protection. The kidney is recognized as a significant source of GDF15 secretion, particularly under conditions of stress [28]. Recent studies have proposed the concept of a “kidney–brain GDF15 axis,” in which kidney-derived GDF15 acts as an endocrine signal to suppress appetite and regulate systemic metabolism via central GFRAL receptors [29]. For instance, metformin has been shown to ameliorate metabolic dysfunction by enhancing renal GDF15 synthesis and secretion, thereby activating central GFRAL signaling [30]. Consistent with this newly discovered regulatory axis, this study found that the levels of GDF15 in the kidneys and serum of DIO-R mice were both elevated. Meanwhile, these mice exhibited weight loss, reduced ectopic lipid deposition in the kidneys, and alleviated kidney injury. Additionally, knocking down GFRAL in obese mice to block the central effect of GDF15 significantly exacerbated the degree of obesity and kidney injury. Based on the existing findings, we propose that the anti-obesity and kidney-protective phenotypes observed in DIO-R mice are at least partially mediated by this kidney–brain GDF15 axis. At the central level, GDF15 binds to the GFRAL receptor in the brain, suppressing appetite and reducing the systemic metabolic burden, thereby indirectly improving lipid homeostasis. At the renal level, GDF15 directly alleviates renal ectopic lipid deposition by enhancing renal autophagy and fatty acid oxidation. The combination of the dual effects at the central and peripheral levels enables GDF15 to coordinately regulate systemic metabolism and renal lipotoxicity.

Ectopic lipid deposition in the kidney and the resulting lipotoxicity are primary causes of oxidative stress, endoplasmic reticulum (ER) stress, and mitochondrial dysfunction, ultimately leading to renal injury [31,32,33]. As a critical intracellular clearance mechanism, autophagy plays a pivotal role in the removal of damaged organelles and lipid droplets [34]. The present study demonstrates that GDF15 significantly enhances autophagy by activating the AMPK/SIRT1 signaling pathway. AMPK, a cellular energy sensor, functions in concert with the NAD+-dependent deacetylase Sirtuin 1 (SIRT1) to regulate metabolism and autophagy [35]. Specifically, AMPK activates SIRT1 by increasing intracellular NAD+ levels, thereby initiating autophagy [36,37]. The results indicate that GDF15 significantly upregulates AMPK phosphorylation and SIRT1 expression; however, the effects were attenuated by the AMPK inhibitor Compound C and the SIRT1 inhibitor EX527. These findings elucidate the molecular pathway by which GDF15 regulates autophagy via the AMPK/SIRT1 axis.

**Figure 7:**
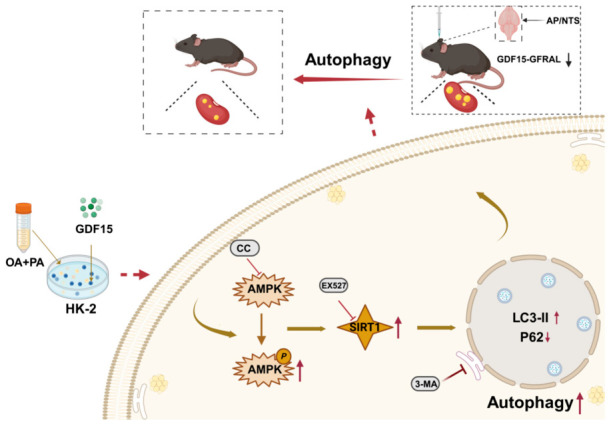
GDF15 activates autophagy via the AMPK/SIRT1 signaling axis, thereby attenuating renal injury consequent to ectopic lipid deposition.

Under a high-fat environment, GDF15 treatment can significantly reduce lipid droplet accumulation and regulate the expression of proteins related to lipid metabolism; in addition, it can enhance the level of autophagy and may indirectly upregulate the expression of CPT-1, thereby promoting the catabolism of fatty acids. However, the protective effects of GDF15 were significantly reversed upon the inhibition of autophagy using 3-MA. These findings indicate that GDF15-induced autophagy is a prerequisite for promoting fatty acid oxidation and alleviating renal lipotoxic injury. This study further validates the mechanism by which GDF15 ameliorates lipotoxicity via the autophagic pathway. In addition to playing a key role in maintaining renal homeostasis, autophagy is also recognized as a protective mechanism against kidney-related diseases [38,39,40]. In summary, this study closely links GDF15, autophagy, and renal lipotoxicity induced by ectopic lipid deposition, thereby elucidating the mechanism underlying the renal protective effects of GDF15 in obesity resistance (Figure 7).

Limitations of the Present Study. Food intake, caloric consumption, and energy expenditure were not assessed in the current study. Future investigations combining measurements of feeding behavior and energy metabolism will provide a more comprehensive understanding of the observed effects. The impact of gut microbial flora on the development of DIO and DIO-R has not been evaluated, which will constitute a key direction for our future research endeavors. Autophagic flux was not directly monitored using lysosomal inhibitors, which represents a methodological limitation of the present study. Future studies using chloroquine or bafilomycin A1 are warranted to further validate autophagic flux. The tissue specificity of AAV-mediated GFRAL knockdown was not experimentally validated in peripheral tissues. Future studies directly examining GFRAL expression in peripheral organs will help confirm the central specificity of the knockdown.

## 5. Conclusions

GDF15 ameliorates renal injury caused by ectopic lipid deposition through activation of the AMPK/SIRT1 signaling pathway, which enhances autophagy and promotes fatty acid oxidation, thereby providing a potential therapeutic target for improving clinical ectopic lipid accumulation and obesity-related glomerulopathy.

## Data Availability

The datasets generated during and/or analyzed during the current study are available from the corresponding author on reasonable request.

## Abbreviations

The following abbreviations are used in this manuscript:

FFA	free fatty acid
Cre	creatinine
BUN	blood urea nitrogen
q-PCR	Quantitative Real-time PCR
IF	Immunofluorescence
AAV	adeno-associated virus
NC	normal control
DIO	diet-induced obesity
DIO-R	diet-induced obesity resistance
HFD	high-fat diet
AMPK	AMP-activated protein kinase
GDF15	Growth differentiation factor 15
TG	triglyceride
ELD	ectopic lipid deposition
ORG	obesity-related glomerulopathy
BCA	bicinchoninic acid

## References

[1] Martínez-Montoro J.I., Morales E., Cornejo-Pareja I., Tinahones F.J., Fernández-García J.C. (2022). Obesity-Related Glomerulopathy: Current Approaches and Future Perspectives. Obes. Rev..

[2] Chen Y., Zhang P., Lv S., Su X., Du Y., Xu C., Jin Z. (2022). Ectopic Fat Deposition and Its Related Abnormalities of Lipid Metabolism Followed by Nonalcoholic Fatty Pancreas. Endosc. Ultrasound.

[3] Janssen J.A.M.J.L. (2024). The Causal Role of Ectopic Fat Deposition in the Pathogenesis of Metabolic Syndrome. Int. J. Mol. Sci..

[4] Dilaver R.G., Afsar R.E., Crescenzi R., Gamboa J., Ikizler T.A. (2025). Effects of Dulaglutide on Ectopic Fat Deposition in Chronic Kidney Disease (CKD): A Pilot and Feasibility Study (GLIMP). medRxiv.

[5] Frampton J., Murphy K.G., Frost G., Chambers E.S. (2020). Short-Chain Fatty Acids as Potential Regulators of Skeletal Muscle Metabolism and Function. Nat. Metab..

[6] Jang S.Y., Choi K.M. (2025). Impact of Adipose Tissue and Lipids on Skeletal Muscle in Sarcopenia. J. Cachexia Sarcopenia Muscle.

[7] Pan X. (2022). The Roles of Fatty Acids and Apolipoproteins in the Kidneys. Metabolites.

[8] Huang T., Wu T., Wu Y., Li X., Tan J., Shen C., Xiong S., Feng Z., Gao S., Li H. (2023). Long-Term Statins Administration Exacerbates Diabetic Nephropathy via Ectopic Fat Deposition in Diabetic Mice. Nat. Commun..

[9] Filali-Mouncef Y., Hunter C., Roccio F., Zagkou S., Dupont N., Primard C., Proikas-Cezanne T., Reggiori F. (2022). The Ménage à Trois of Autophagy, Lipid Droplets and Liver Disease. Autophagy.

[10] Meçe O., Houbaert D., Sassano M.-L., Durré T., Maes H., Schaaf M., More S., Ganne M., García-Caballero M., Borri M. (2022). Lipid Droplet Degradation by Autophagy Connects Mitochondria Metabolism to Prox1-Driven Expression of Lymphatic Genes and Lymphangiogenesis. Nat. Commun..

[11] Johnen H., Lin S., Kuffner T., Brown D.A., Tsai V.W.-W., Bauskin A.R., Wu L., Pankhurst G., Jiang L., Junankar S. (2007). Tumor-Induced Anorexia and Weight Loss Are Mediated by the TGF-β Superfamily Cytokine MIC-1. Nat. Med..

[12] Yang L., Chang C.-C., Sun Z., Madsen D., Zhu H., Padkjær S.B., Wu X., Huang T., Hultman K., Paulsen S.J. (2017). GFRAL Is the Receptor for GDF15 and Is Required for the Anti-Obesity Effects of the Ligand. Nat. Med..

[13] Mullican S.E., Lin-Schmidt X., Chin C.-N., Chavez J.A., Furman J.L., Armstrong A.A., Beck S.C., South V.J., Dinh T.Q., Cash-Mason T.D. (2017). GFRAL Is the Receptor for GDF15 and the Ligand Promotes Weight Loss in Mice and Nonhuman Primates. Nat. Med..

[14] Cocozza G., Busdraghi L.M., Chece G., Menini A., Ceccanti M., Libonati L., Cambieri C., Fiorentino F., Rotili D., Scavizzi F. (2025). GDF15-GFRAL Signaling Drives Weight Loss and Lipid Metabolism in Mouse Model of Amyotrophic Lateral Sclerosis. Brain. Behav. Immun..

[15] Mazagova M., Buikema H., Van Buiten A., Duin M., Goris M., Sandovici M., Henning R.H., Deelman L.E. (2013). Genetic Deletion of Growth Differentiation Factor 15 Augments Renal Damage in Both Type 1 and Type 2 Models of Diabetes. Am. J. Physiol. Renal Physiol..

[16] Li Y., Chen Y., Qin Z.-H. (2019). AMPK and Autophagy. Autophagy: Biology and Diseases.

[17] Aguilar-Recarte D., Barroso E., Gumà A., Pizarro-Delgado J., Peña L., Ruart M., Palomer X., Wahli W., Vázquez-Carrera M. (2021). GDF15 Mediates the Metabolic Effects of PPARβ/δ by Activating AMPK. Cell Rep..

[18] Yu Q., Zou L., Yuan X., Fang F., Xu F. (2021). Dexmedetomidine Protects against Septic Liver Injury by Enhancing Autophagy through Activation of the AMPK/SIRT1 Signaling Pathway. Front. Pharmacol..

[19] Sun Y., Xia M., Yan H., Han Y., Zhang F., Hu Z., Cui A., Ma F., Liu Z., Gong Q. (2018). Berberine Attenuates Hepatic Steatosis and Enhances Energy Expenditure in Mice by Inducing Autophagy and Fibroblast Growth Factor 21. Br. J. Pharmacol..

[20] Ren L., Cui H., Wang Y., Ju F., Cai Y., Gang X., Wang G. (2023). The Role of Lipotoxicity in Kidney Disease: From Molecular Mechanisms to Therapeutic Prospects. Biomed. Pharmacother..

[21] Keipert S., Ost M. (2021). Stress-Induced FGF21 and GDF15 in Obesity and Obesity Resistance. Trends Endocrinol. Metab..

[22] Lasaad S., Crambert G. (2024). GDF15, an Emerging Player in Renal Physiology and Pathophysiology. Int. J. Mol. Sci..

[23] Wang D., Townsend L.K., DesOrmeaux G.J., Frangos S.M., Batchuluun B., Dumont L., Kuhre R.E., Ahmadi E., Hu S., Rebalka I.A. (2023). GDF15 Promotes Weight Loss by Enhancing Energy Expenditure in Muscle. Nature.

[24] Emmerson P.J., Wang F., Du Y., Liu Q., Pickard R.T., Gonciarz M.D., Coskun T., Hamang M.J., Sindelar D.K., Ballman K.K. (2017). The Metabolic Effects of GDF15 Are Mediated by the Orphan Receptor GFRAL. Nat. Med..

[25] Tsai V.W.W., Husaini Y., Sainsbury A., Brown D.A., Breit S.N. (2018). The MIC-1/GDF15-GFRAL Pathway in Energy Homeostasis: Implications for Obesity, Cachexia, and Other Associated Diseases. Cell Metab..

[26] Zhu C., Liu Q., Su Y., Zhang Y., Patel A., Greasley A., Jiang J., Quan D., Min W., Liu K. (2025). Overexpression of GDF15 Protects Kidneys from Ischemia Reperfusion Injury and Affects Circular RNA Expression. Front. Cell Dev. Biol..

[27] Mori Y., Ajay A.K., Chang J.-H., Mou S., Zhao H., Kishi S., Li J., Brooks C.R., Xiao S., Woo H.-M. (2021). KIM-1 Mediates Fatty Acid Uptake by Renal Tubular Cells to Promote Progressive Diabetic Kidney Disease. Cell Metab..

[28] Tang Y., Liu T., Sun S., Peng Y., Huang X., Wang S., Zhou Z. (2024). Role and Mechanism of Growth Differentiation Factor 15 in Chronic Kidney Disease. J. Inflamm. Res..

[29] Garrido A.N., Zhang S.-Y., Bruce K., Lai C.S.H., Yang Z., Wang M.T., Lam T.K.T. (2025). Lipids Engage a Kidney-Brain GDF15 Axis to Suppress Food Intake. Diabetes.

[30] Zhang S.-Y., Bruce K., Danaei Z., Li R.J.W., Barros D.R., Kuah R., Lim Y.-M., Mariani L.H., Cherney D.Z., Chiu J.F.M. (2023). Metformin Triggers a Kidney GDF15-Dependent Area Postrema Axis to Regulate Food Intake and Body Weight. Cell Metab..

[31] Nishi H., Higashihara T., Inagi R. (2019). Lipotoxicity in Kidney, Heart, and Skeletal Muscle Dysfunction. Nutrients.

[32] Li Y.-X., Han T.-T., Liu Y., Zheng S., Zhang Y., Liu W., Hu Y.-M. (2015). Insulin Resistance Caused by Lipotoxicity Is Related to Oxidative Stress and Endoplasmic Reticulum Stress in LPL Gene Knockout Heterozygous Mice. Atherosclerosis.

[33] Zhang X., Agborbesong E., Li X. (2021). The Role of Mitochondria in Acute Kidney Injury and Chronic Kidney Disease and Its Therapeutic Potential. Int. J. Mol. Sci..

[34] Maestri A., Garagnani P., Pedrelli M., Hagberg C.E., Parini P., Ehrenborg E. (2024). Lipid Droplets, Autophagy, and Ageing: A Cell-Specific Tale. Ageing Res. Rev..

[35] Cantó C., Gerhart-Hines Z., Feige J.N., Lagouge M., Noriega L., Milne J.C., Elliott P.J., Puigserver P., Auwerx J. (2009). AMPK Regulates Energy Expenditure by Modulating NAD+ Metabolism and SIRT1 Activity. Nature.

[36] Price N.L., Gomes A.P., Ling A.J.Y., Duarte F.V., Martin-Montalvo A., North B.J., Agarwal B., Ye L., Ramadori G., Teodoro J.S. (2012). SIRT1 Is Required for AMPK Activation and the Beneficial Effects of Resveratrol on Mitochondrial Function. Cell Metab..

[37] Xu T., Song Q., Zhou L., Yang W., Wu X., Qian Q., Chai H., Han Q., Pan H., Dou X. (2021). Ferulic Acid Alleviates Lipotoxicity-Induced Hepatocellular Death through the SIRT1-Regulated Autophagy Pathway and Independently of AMPK and Akt in AML-12 Hepatocytes. Nutr. Metab..

[38] Tang C., Livingston M.J., Liu Z., Dong Z. (2020). Autophagy in Kidney Homeostasis and Disease. Nat. Rev. Nephrol..

[39] Liu X., Jiang L., Zeng H., Gao L., Guo S., Chen C., Liu X., Zhang M., Ma L., Li Y. (2024). *Circ-0000953* Deficiency Exacerbates Podocyte Injury and Autophagy Disorder by Targeting *Mir665-3p-Atg4b* in Diabetic Nephropathy. Autophagy.

[40] Yang W., Jia M., Zhou F., Zhang M., Qu S., Zeng C., Zhu X., Gu S., Xuan J., Liu Z. (2025). Renal Tubular VMP1 Protects against Acute Kidney Injury via Modulating Autophagy and Autophagy-Independent Pathway. Autophagy.

